# Supplementary material for: Automated data extraction from historical city directories: The rise and fall of mid-century gas stations in Providence, RI
Source: PLoS One. 2020 Aug 19;15(8):e0220219. doi: 10.1371/journal.pone.0220219 (PMC7437912; doi:10.1371/journal.pone.0220219)
Supplement: Attachment — Submitted filename: ResponseToReviewer.docx [file pone.0220219.s001.docx]

I have also read your paper and I like it. Permit me to share a few thoughts.

Thank you!

The paper addresses an important roadblock in research questions investigating historical microdata, the time-consuming task of transcribing large amounts of data. In the past, this arduous task has been delegated to research assistants, interns, and more recently crowdsourced through “digital volunteers” or “micro-volunteers”. The paper also highlights the issue of the toxic legacy of buildings prior to 1976, which may be unknown as land use patterns change over time.

A good summary of the purpose of this work.

The methods laid out in the paper are sound and should address the majority of steps needed to process historical directories. However, a main question is: how viable is the method for smaller numbers of records? For under 2,500 records, a research assistant coder could probably complete the same task in a few days.

For very small numbers of records, of course, much of this could be done by hand. However, hand work is exceptionally tedious, and as a result of the tedium, it is also prone to errors. Moreover, the lack of easy reproducibility makes it difficult to make changes to the methodological choices, locking hand studies into the decisions made initially before more mature reflection. Once one gets to the scale of a book, hand processing becomes ineffective. We certainly do agree that there is a limit to how much should be automated. At a certain point, it can be more work to automate everything. For instance, we have allowed for minimal human interference in the image processing steps.

There are a variety of ways that errors could enter the processing pipeline, leading to necessary double checking that would be duplicative of the work of coders.

The way we look at it, human examination of the data is an important part of coding. It is important not to get lost in the code, allowing hidden errors to slip through.

The larger the dataset and more years investigated, the more this method becomes viable.

Agreed.

Image quality seems to be a major factor in the utility of this method and good quality scans of well-maintained historical documents are crucial.

Yes. However, this is also true for other methods, such as human examination.

The development of this method is promising and demonstrates the future possibilities and efficacy of machine learning in converting historical paper databases to useful microdata for researchers.

We hope so!

In your revision, please try to incorporate the reviewer's points and my discussion. I look forward to reading your revision.

1. Is the manuscript technically sound, and do the data support the conclusions?

The manuscript must describe a technically sound piece of scientific research with data that supports the conclusions. Experiments must have been conducted rigorously, with appropriate controls, replication, and sample sizes. The conclusions must be drawn appropriately based on the data presented. 

Reviewer #1: Partly

2. Has the statistical analysis been performed appropriately and rigorously? 

Reviewer #1: N/A

3. Have the authors made all data underlying the findings in their manuscript fully available?

The [PLOS Data policy](http://www.plosone.org/static/policies.action#sharing) requires authors to make all data underlying the findings described in their manuscript fully available without restriction, with rare exception (please refer to the Data Availability Statement in the manuscript PDF file). The data should be provided as part of the manuscript or its supporting information, or deposited to a public repository. For example, in addition to summary statistics, the data points behind means, medians and variance measures should be available. If there are restrictions on publicly sharing data—e.g. participant privacy or use of data from a third party—those must be specified.

Reviewer #1: Yes

4. Is the manuscript presented in an intelligible fashion and written in standard English?

PLOS ONE does not copyedit accepted manuscripts, so the language in submitted articles must be clear, correct, and unambiguous. Any typographical or grammatical errors should be corrected at revision, so please note any specific errors here.

Reviewer #1: Yes

5. Review Comments to the Author

Please use the space provided to explain your answers to the questions above. You may also include additional comments for the author, including concerns about dual publication, research ethics, or publication ethics. (Please upload your review as an attachment if it exceeds 20,000 characters)

Reviewer #1: I enjoyed this article and think that the process you’re describing has broad utility for social science researchers, and any other researchers that might want to rely on old text as data.

Great!

A. Validation

A key statistic in this paper is that about 95 percent of gas stations (94.6) are “correctly identified.” I was never entirely sure what this term meant, and that the baseline for “correct” was. On page 16, I think we learn that “success” does not mean that your method returns a result that is as good as hand-coding – which I think is the most natural definition of success. As far as I understand, “success” means that the processing runs without errors.

This is fair. We have clarified our definition of “correctly identified.” The main type of error that we excluded was OCR errors in the business names.

More generally, I think the paper would be tremendously stronger if you more generally considered
- true positives
- false positives
in addition to what you do already quite well, which is consider
- true negatives
- false negatives
Suppose you geocode an address “successfully,” but that location is wrong. This is a false positive. Do you have any sense of the scope of false positives in your data? You do a good job looking at negatives – I think the paper would be strengthened by looking at positives as well.

We have clarified this better in the text. Actually, we did identify one case of what we consider a false positive, where an address was incorrectly read as another address and successfully geocoded. We lumped this into the overall accuracy rate. We have added a section exploring the issue of false positives and highlighting exactly what happened in the one false positive case in our hand validation sample.

We deliberately designed our algorithm to minimize these false positives, and we believe it is very important to avoid them. Before we required a geocoder score of 100, we had a lager (but still small) number of cases where we could identify false positives. For instance, we identified a case where a street had been partially destroyed, and the geocoder placed an address at the end of the existing street with a lower geocoder score. By requiring a score of 100, the geocoder will only match when the address exactly matches an address in its database. However, there are a few cases where we may still get rare false positives, as we discuss in a section we have added.

At multiple places in section 3, you write some version of “things fail and require correction by hand.” It would be very helpful to know
- how many times you had to do this
- what “fails” means. Does this mean there was an error and the image wouldn’t process? Or that you looked at the output and decided it was of too low quality? If the latter, what was your metric?

We explore this in the first paragraph of our results section. We felt that it was better to think of the rate of hand-correction needed from the image processing pipeline as a result, rather than part of the methods section.

We removed some confusing sections of the grayscale thresholding section describing how we built an option for hand adjustment of the grayscale data that gave off the incorrect impression that we had gone through the hand-adjusting process for these data.

What do you do with the addresses that fail to match? Do you just leave them as unmatched? Or do you follow-up with hand coding?

We added two sentences to clarify that we do not hand-code addresses that fail to match, but *directoreadr* does output a file containing all the failed matches that can be examined by hand.

On page 17, you write that “the geocoder algorithm produced errors in 4.7% of cases.” What is an error here? Where the program fails to code at all? Is a location outside Providence an error? And what about incorrect coding of addresses that are coded?

We added a clarification that errors are thrown whenever the geocoder does not produce a perfect confidence score. If it has a location with less than absolute confidence, we called it a geocoder error. We did this to prevent against false positives, as we say above. A location outside Providence will nearly always throw an error, unless we have mistakenly identified it as a street in Providence, and it is a common address name that is also present in Providence. While we did not encounter any examples of this happening in our hand checking, it is definitely possible that it happened in a very limited number of case.

It would also be helpful to show the rate of failure by year (not the number, but the rate). This would help readers assess whether this technique is reasonable for time series data.

Figure 2 does show rate and not absolute number, and it includes the geocoder failure rate as well as the address drop rate. We have kept this feature in the reworked figure.

B. And what do you do with this?

At the end of the article, you give some tantalizing short sentences on how the number of gas stations has declined tremendously over time in Providence. It would be extremely interesting to understand the determinants of gas station depth.

The idea here was to focus this paper on the methods, with gas stations really only being an example application. Further papers will explore the applications.

C. Visualizations

You present a number of over-time charts. When showing data over time, it is both standard and helpful to use line charts. In your case, I’d use a line with dots; the dots will indicate where you actually have data, and the lines will allow us to follow the key patterns over time.

We agree with this suggestion and have remade Figure 2 along these lines.

D. Writing

I found the overall organization and communication in this paper to be quite sound.

The writing more broadly would be substantially improved with the help of a professional editor. These edits would go a long way toward improving the readability of the paper and get your points across more effectively.

We have re-edited the paper and made changes to improve readability of the text.
